# A Diverse Virome Is Identified in Parasitic Flatworms of Domestic Animals in Xinjiang, China

**DOI:** 10.1128/spectrum.00702-23

**Published:** 2023-04-12

**Authors:** Peng Zhang, Yao Zhang, Le Cao, Jun Li, Chuanchuan Wu, Mengxiao Tian, Zhuangzhi Zhang, Chiyu Zhang, Wenbao Zhang, Yanpeng Li

**Affiliations:** a Shanghai Public Health Clinical Center, Fudan University, Shanghai, China; b State Key Laboratory of Pathogenesis, Prevention and Treatment of High Incidence Diseases in Central Asia, WHO-Collaborating Centre for Prevention and Care Management of Echinococcosis, Xinjiang Medical University, The First Affiliated Hospital of Xinjiang Medical University, Urumqi, Xinjiang, China; c Veterinary Research Institute, Xinjiang Academy of Animal Sciences, Urumqi, Xinjiang, China; Changchun Veterinary Research Institute

**Keywords:** virome, parasitic flatworms, virus evolution, viral metagenomics, metagenomics, virus discovery

## Abstract

Parasitic flatworms infect diverse vertebrates and are major threats to animal and even human health; however, little is known about the virome of these lower life forms. Using viral metagenomic sequencing, we characterized the virome of the parasitic flatworms collected from major domestic animals, including Dicrocoelium lanceatum and Taenia hydatigena, Echinococcus granulosus *sensu stricto* and Echinococcus multilocularis. Seven and three different viruses were discovered from *D. lanceatum* and *T. hydatigena*, respectively, and no viral sequences were found in adult tapeworms and protoscoleces of *E. granulosus sensu stricto* and E. multilocularis. Two out of the five parasitic flatworm species carry viruses, showing a host specificity of these viruses. These viruses belong to the *Parvoviridae*, *Circoviridae*, unclassified circular, Rep-encoding single-stranded (CRESS) DNA virus, *Rhabdoviridae*, *Endornaviridae*, and unclassified RNA viruses. The presence of multiple highly divergent RNA viruses, especially those that cluster with viruses found in marine animals, implies a deep evolutionary history of parasite-associated viruses. In addition, we found viruses with high identity to common pathogens in dogs, including canine circovirus and canine parvovirus 2. The presence of these viruses in the parasites implies that they may infect parasitic flatworms but does not completely exclude the possibility of contamination from host intestinal contents. Furthermore, we demonstrated that certain viruses, such as CRESS DNA virus may integrate into the genome of their host. Our results expand the knowledge of viral diversity in parasites of important domestic animals, highlighting the need for further investigations of their prevalence among other parasites of key animals.

**IMPORTANCE** Characterizing the virome of parasites is important for unveiling the viral diversity, evolution, and ecology and will help to understand the “Russian doll” pattern among viruses, parasites, and host animals. Our data indicate that diverse viruses are present in specific parasitic flatworms, including viruses that may have an ancient evolutionary history and viruses currently circulating in parasite-infected host animals. These data also raise the question of whether parasitic flatworms acquire and/or carry some viruses that may have transmission potential to animals. In addition, through the study of virus-parasite-host interactions, including the influence of viral infection on the life cycle of the parasite, as well as its fitness and pathogenicity to the host, we could find new strategies to prevent and control parasitic diseases.

## INTRODUCTION

Viruses are the most abundant but understudied components of the entire biodiversity, replicating in almost all host organisms (1, 2). The last few decades have witnessed outbreaks of emerging and/or reemerging viruses that have massive health, social, and economic impacts, e.g., Ebola virus, H1N1 influenza virus, Zika virus, and SARS-CoV-2, highlighting a global vulnerability to emerging viral diseases (3, 4). It is therefore important to conduct virus discovery and surveillance of the global virome, unveil the virus diversity and ecology, identify novel species or variants of existing pathogenic viruses, and investigate the transmission history or evolutionary trajectory of the viruses that pose public health concerns for humans or animals. Despite the explosive efforts dedicated to exploring the immense virosphere (5, 6), current knowledge of the global virome remains limited or biased, with most studies focusing on chordates, such as mammals and birds, and arthropods, including mosquitoes and ticks (7, 8).

Parasitic flatworms constitute diverse species and have high prevalence in both humans and livestock (9, 10). Parasitic flatworms and their associated diseases are difficult to target and treat, as parasitic infections can be asymptomatic and misdiagnosed (11, 12). Even though current knowledge of viruses in flatworms is still limited, several studies have shown the presence of different viruses in these lower life forms. The first evidence of viruses in flatworms was identified as early as the 1960s to 1970s, with reports of viral-like particles in the apicomplexan and kinetoplastid phyla (13, 14). Since then, increasing evidence has characterized flatworm-associated viruses, including double-stranded RNA (dsRNA) viruses in the human parasites Giardia, *Leishmania*, *Trichomonas*, and *Cryptosporidium* (15–18); single-stranded DNA (ssDNA) viruses, a large nidovirus, and a new family of toti-like viruses were also found in free-living flatworms (19–21). In addition, single-stranded *Narnavirus*-like RNA viruses were identified in Leptomonas seymouri (22, 23) and Plasmodium vivax (24) in human malaria. Viruses from the *Bunyavirales* and *Nyamiviridae* have been reported in Schistosoma japonicum and *Taenia* spp. (2). Schistocephalus solidus was reported to carry multiple viruses, including rhabdovirus, nyamivirus, jingchuvirus, bunya-like virus, and toti-like virus (25). Most recently, through a data mining approach, 115 viral sequences were discovered from different Platyhelminthes-related sequences in the public databases (8), indicating that the distribution of viruses in parasites is a common phenomenon.

This so-called Russian doll infection (parasites are themselves infected by other microbes) is an interesting phenomenon (26), as the interactions between viruses and parasites may in turn impact the fitness, virulence, and pathogenesis of the parasite (27). For example, the infection of Leishmania guyanensis and Trichomonas vaginalis by *Leishmania* RNA virus 1 (LRV1) and Trichomonas vaginalis virus, respectively, would promote parasite pathogenesis (28, 29), and parasites harboring viruses are also associated with treatment failure in patients with leishmaniasis (30, 31). Even though the underlying mechanisms of how these viruses influence the fitness of parasites and, subsequently, host diseases are not fully clear, previous evidence has suggested a possible way through the stimulation of the host proinflammatory response (32, 33). A recent study showed that virus could be vertically transmitted and persist throughout the life cycle of the parasite, as well as the transmission between different hosts (25).

Domestic animals are the reservoirs for many parasites, and parasitic diseases are major threats to the health of these animals as well as the global animal husbandry industry (8, 34–36). However, whether these parasites carry known or novel viruses and have an impact on parasite biology and pathogenesis remains largely unknown. In this study, we aim to investigate the virome of the major parasitic flatworms Echinococcus granulosus *sensu stricto* and Taenia hydatigena living with sheep and dogs as intermediate and definitive hosts, respectively, and Dicrocoelium lanceatum in sheep liver. Echinococcosis, cysticercosis tenuicollis, and dicroceliasis caused by these helminths are important parasitic diseases in farm animals in Xinjiang, China. The knowledge of basic virus-parasite biology will provide novel insights into parasitic infections in these animals and may unravel new strategies to prevent and control parasitic diseases.

## RESULTS

### Overview of parasite-associated viruses.

**FIG 1:**
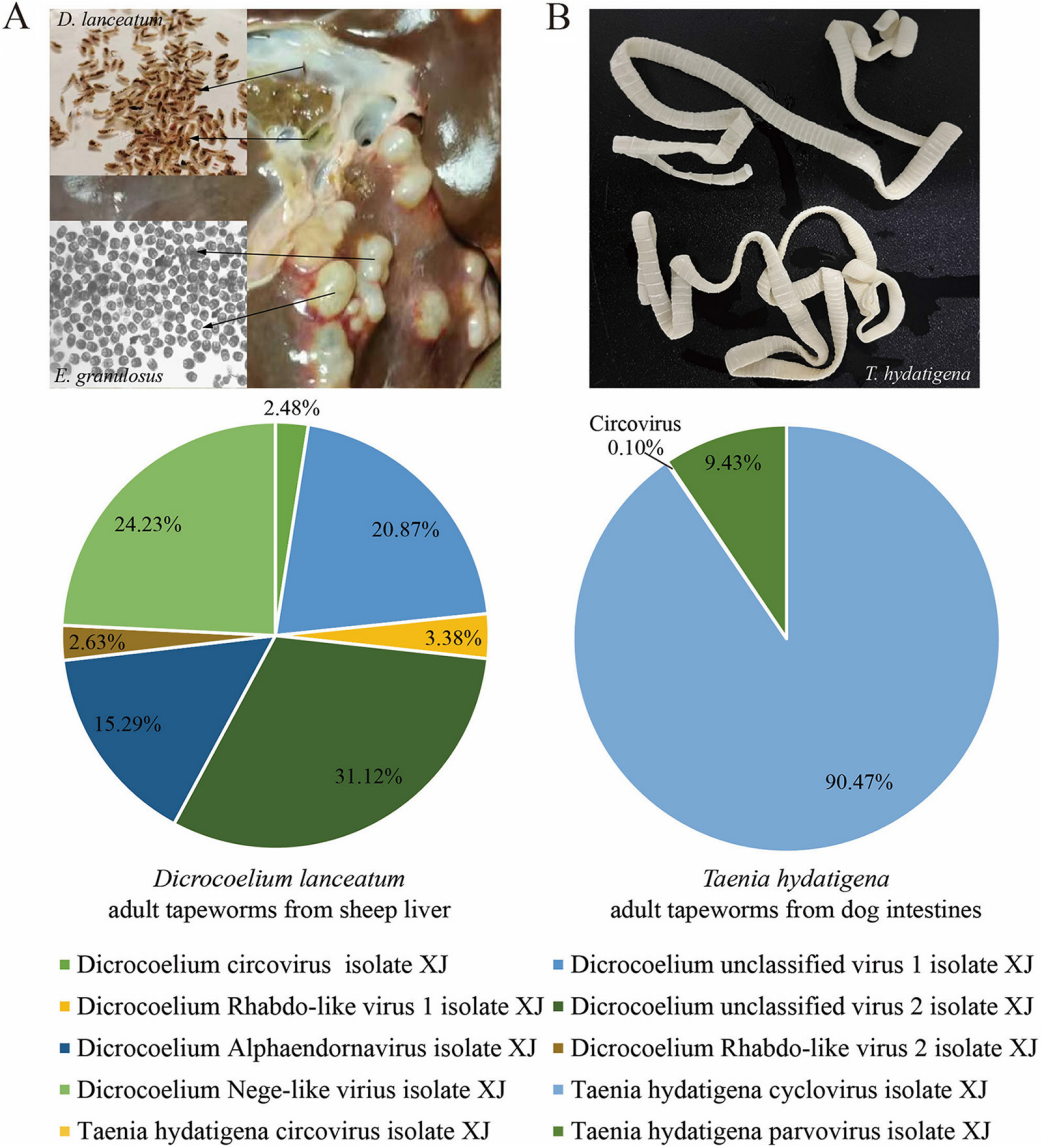
Overview of all the viruses in the different sample pools of parasites. (A) Pictures of *D. lanceatum* and *E. granulosus* isolated from the same sheep liver. (B) Pictures of *T. hydatigena* isolated from dog intestine. The relative abundance of different eukaryotic viruses in adult flukeworms of *D. lanceatum* from sheep liver (A) and *T. hydatigena* from dog intestines (B) is shown. No viral sequences were detected in the sample pools of *E. granulosus* from wither sheep liver and dog intestine or from sample pools of E. multilocularis from dog intestine.

During May to August of 2021, four species of main tapeworms (*E. granulosus sensu stricto*, Echinococcus multilocularis, *T. hydatigena* and *D. lanceatum*) were collected from their corresponding host animals from Urumuqi, Xinjiang, China (see Materials and Methods). Metagenomic sequencing was performed on 21 libraries, containing 46 adult worms (10 *E. granulosus sensu stricto*, 10 E. multilocularis, 16 *T. hydatigena* tapeworms, and 10 *D. lanceatum* fluke worms) and 2,000 protoscoleces (larval stage of *E. granulosus sensu stricto* and E. multilocularis; Table 1). In total, 344.7 million paired-end sequence reads (ranging from 9.23 to 24.16 million; median of 16.28 million sequences per sample) were generated. After quality control, there were 259 million clean reads (range, 6.39 to 20.87 million; median, 11.70 million). Following *de novo* assembly, all the sequences were annotated using the virus-only nucleotide and protein databases (see Materials and Methods). In total, 0.13 million (0.05% of clean reads) sequences were annotated as eukaryotic viral origins. Eukaryotic viral sequences were identified in adult fluke worms of *D. lanceatum* from sheep liver and adult tapeworms of *T. hydatigena* from dog intestines, and no viral sequences were found in other libraries, including adult tapeworms and protoscoleces of *E. granulosus sensu stricto* and E. multilocularis (Fig. 1). All these viral sequences belong to *Rhabdoviridae*, *Endornaviridae*, *Parvoviridae*, *Circoviridae*, unclassified circular viruses, and unclassified RNA viruses.

**TABLE 1:** Sample information of the parasitic flatworms collected from animals

Animal	Organ	Species of flatworm	Stage of life cycle	No. of individuals
Sheep	Liver	*Dicrocoelium lanceatum*	Adult flukeworms	10
	Liver	*Echinococcus granulosus*	Protoscoleces	1,000
Dog	Intestines	*Echinococcus granulosus*	Adult tapeworms	10
	Intestines	Echinococcus multilocularis	Adult tapeworms	10
	Intestines	*Taenia hydatigena*	Adult tapeworms	16
Mouse	Liver	Echinococcus multilocularis	Protoscoleces	1,000

Details of these viral sequences, including contigs, the best-hit in the database, genetic identity to the best-hit, and genome coverage are listed in Table 2. *D. lanceatum* contained diverse viruses, including three unclassified RNA viruses, which accounted for 31.1%, 24.2%, and 20.9% of the viral abundance, respectively. Other viruses in *D. lanceatum* include circular, Rep-encoding single-stranded (CRESS) DNA virus, *Alphaendornavirus*, and two Rhabdo-like viruses (Fig. 1A). The viruses in *T. hydatigena* were dominated by cyclovirus (90.5%), and parvovirus (9.4%) and circovirus (0.1%) were low in abundance (Fig. 1B).

**TABLE 2:** Summary of the eukaryotic viral sequences retrieved from different parasitic flatworms

Species of flatworm	Virus detected	Contig length (bp) (longest)	Best blastn hit of the longest contig	Contig identity (%)	Genome coverage (%)
*Dicrocoelium lanceatum* adult flukeworms from sheep liver	*Dicrocoelium* unclassified virus 1 isolate XJ	13,069	MW452295	46.64	100
	*Dicrocoelium* circovirus isolate XJ	2,364	KJ641732	49.1	100
	*Dicrocoelium* Rhabdo-like virus 1 isolate XJ	11,778	MG600017	56.61	100
	*Dicrocoelium* unclassified virus 2 isolate XJ	12,895	MW452295	44.21	100
	*Dicrocoelium Alphaendornavirus* isolate XJ	13,889	MW349899	29.95	100
	*Dicrocoelium* Rhabdo-like virus 2 isolate XJ	12,501	BK059698	46.99	100
	*Dicrocoelium* Nege-like virus isolate XJ	9,354	NC032442	57.69	100
*Taenia hydatigena* adult tapeworms from dog intestines	*Taenia hydatigena* cyclovirus isolate XJ	515	KM017740	67.25	49.52
	*Taenia hydatigena* circovirus isolate XJ	278	MZ826144	99.64	13.48
	*Taenia hydatigena* parvovirus isolate XJ	4,869	MH476588	98.94	96.38

### Rhabdo-like virus.

**FIG 2:**
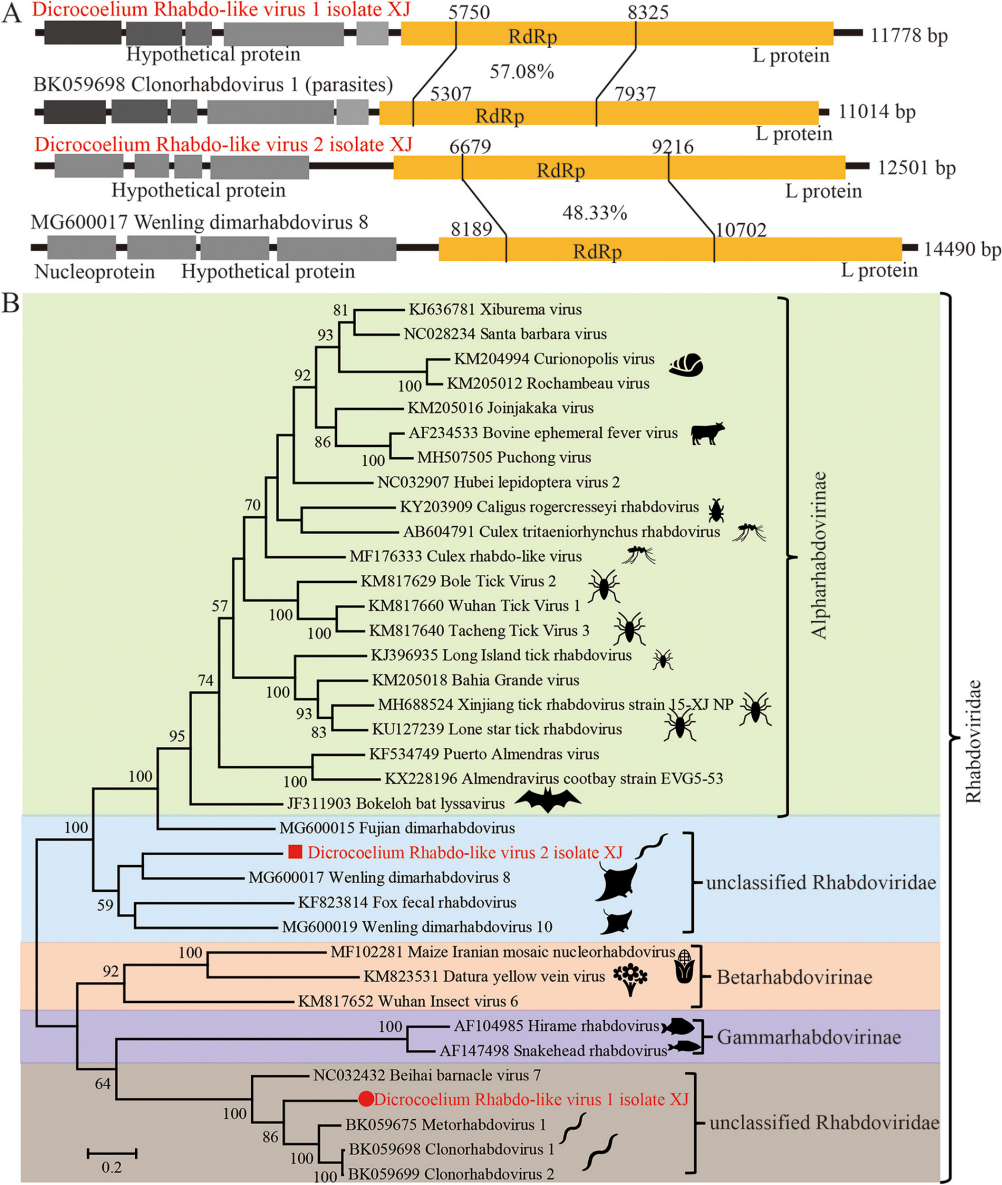
The genomic structures and evolutionary relationships of the newly discovered Rhabdo-like virus (DRLV1/2) with representative viruses from the *Rhabdoviridae.* (A) The genomic structures of DRLV1/2 and its closely related homolog. Hypothetical ORFs, the L protein including the RdRp region, and genetic identities of the RdRp region between DRLV1/2 and its homolog are shown. (B) Phylogenetic trees were generated using the full-length coding sequences of RdRp. MEGA 7 was used for phylogeny inference using the maximum-likelihood (ML) method based on the LG+G model, and the analyses were conducted with 1,000 bootstrap replicates. Representative viruses from different genera of *Rhabdoviridae* and the most closely related viruses from the database were included.

*Rhabdoviridae* is a diverse family of enveloped RNA viruses, with a negative-sense, single-stranded RNA (–ssRNA) genome encoding five typical structural proteins (N, P, M, G, and L) (37–39). The members of the *Rhabdoviridae* infect a wide range of hosts, including plants, vertebrates, and/or invertebrates. *Rhabdoviridae*-related sequences were detected in the adult fluke worms of *D. lanceatum* from sheep liver, and two near-complete genomes of 11,778 and 12,501 bp were successfully assembled: *Dicrocoelium* Rhabdo-like virus 1 isolate XJ (*DiRLV1*, GenBank no. OP548620) and *Dicrocoelium* Rhabdo-like virus 2 isolate XJ (*DiRLV2*, GenBank no. OP627658) (Fig. 2A). Similar to other rhabdoviruses, *DiRLV1/2* contain five or six open reading frames (ORFs), including the largest ORF (L gene) encoding polymerase protein and several small ORFs, possibly encoding N, P, M, and G proteins (Fig. 2A). A phylogenetic tree was constructed based on the conserved RNA-dependent RNA polymerase (RdRp) of *DRhaV1/2* and all the representative viruses from *Rhabdoviridae* (Fig. 2B). *DiRLV1* is most closely related to Clonorhabdovirus 1 from Clonorchis sinensis (BK059698), sharing an RdRp nucleotide identity of 57.1% and a full-length nucleotide identity of 54.1% between the two viruses. *DiRLV2* has an RdRp identity of 48.3% and a full-length identity of 48.3% with the closest relative, Wenling dimarhabdovirus 8 from Okamejei acutispina (MG600017). Currently, there are three subfamilies (alpha-, beta-, and gamma-) of *Rhabdoviridae*, containing 40 genera as well as an unclassified genus. Based on the phylogenetic relationship and the current demarcation criteria, *DiRLV1* and *2* are well supported as novel unclassified species and genera, respectively.

### Alphaendornavirus.

**FIG 3:**
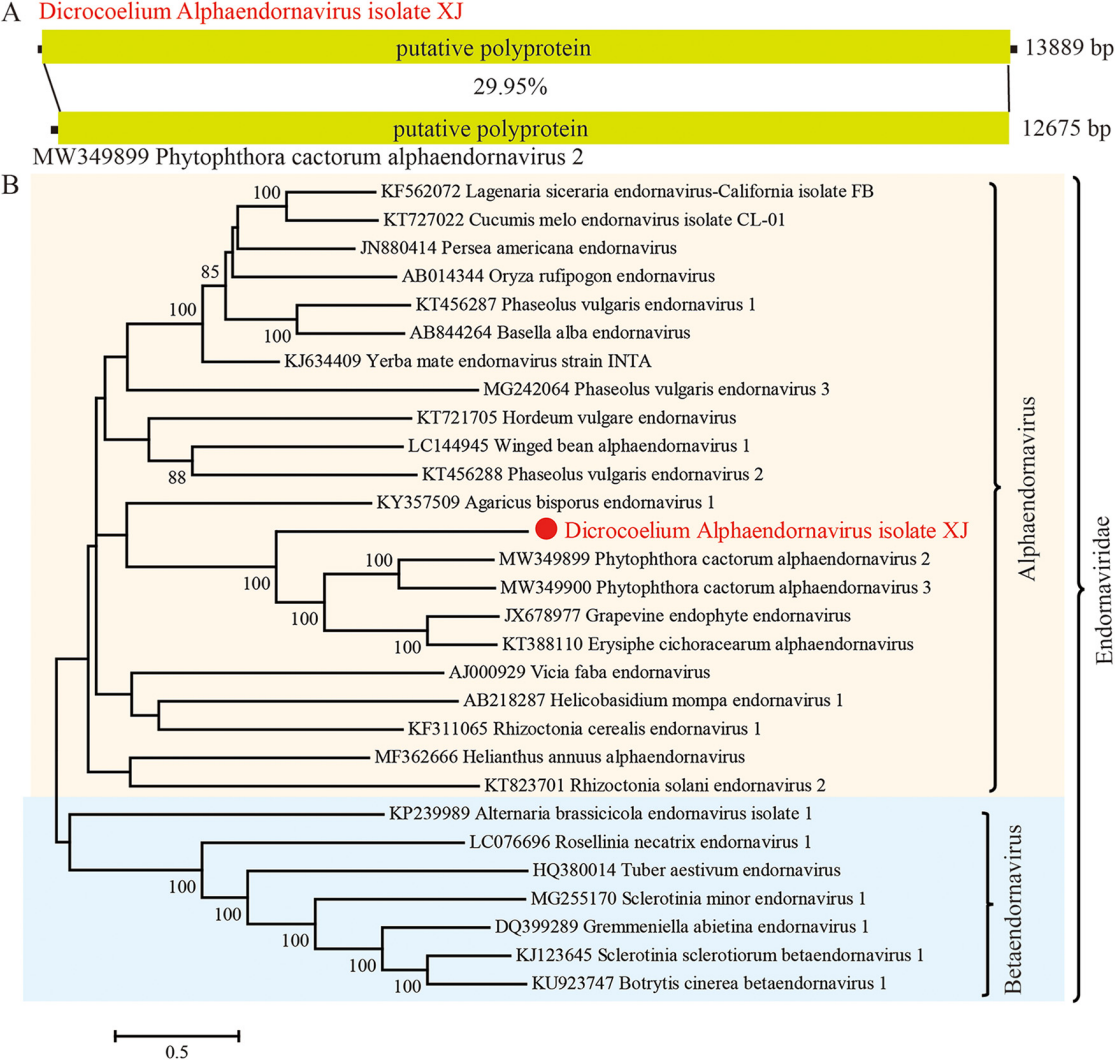
The genomic structures and evolutionary relationships of the newly discovered *Alphaendornavirus* (DiEV) with representative viruses from the *Endornaviridae.* (A) The genomic structures of DiEV and its closely related homolog. Hypothetical ORF and genetic identities of ORFs between DiEV and its homolog are shown. (B) Phylogenetic trees were generated using the full-length coding sequences of ORFs. MEGA 7 was used for phylogeny inference using the maximum-likelihood (ML) method based on the GTR+G+I model, and the analyses were conducted with 1,000 bootstrap replicates. Representative viruses from different genera of *Endornaviridae* and the most closely related viruses from the database were included.

*Endornaviridae* includes viruses with a linear, single-stranded, positive-sense RNA genome ranging from 9.7 to 17.6 kb, which contains a single ORF encoding a polyprotein. These viruses have been reported to infect plants, plant pathogenic fungi, and oomycetes (40) and were classified into *Alphaendornavirus* and *Betaendornavirus*. Sequences related to *Endornaviridae* were detected in the adult flukeworms of *D. lanceatum* from sheep liver, and after assembly, a full-length genome of 13,889 bp was retrieved (Fig. 3A). This virus (*Dicrocoelium Alphaendornavirus*, DiEV, GenBank no. OP548618) encodes a single ORF, sharing only 30% identity of the full genome or 49.8% identity of the RdRp region to its closest relative, Phytophthora cactorum alphaendornavirus 2 (MW349899) (Fig. 3B). The low genetic identity of DiEV to existing endornaviruses indicates the diverse nature of these viruses and a potential independent evolutionary history of DiEV in parasites.

### Unclassified RNA viruses.

**FIG 4:**
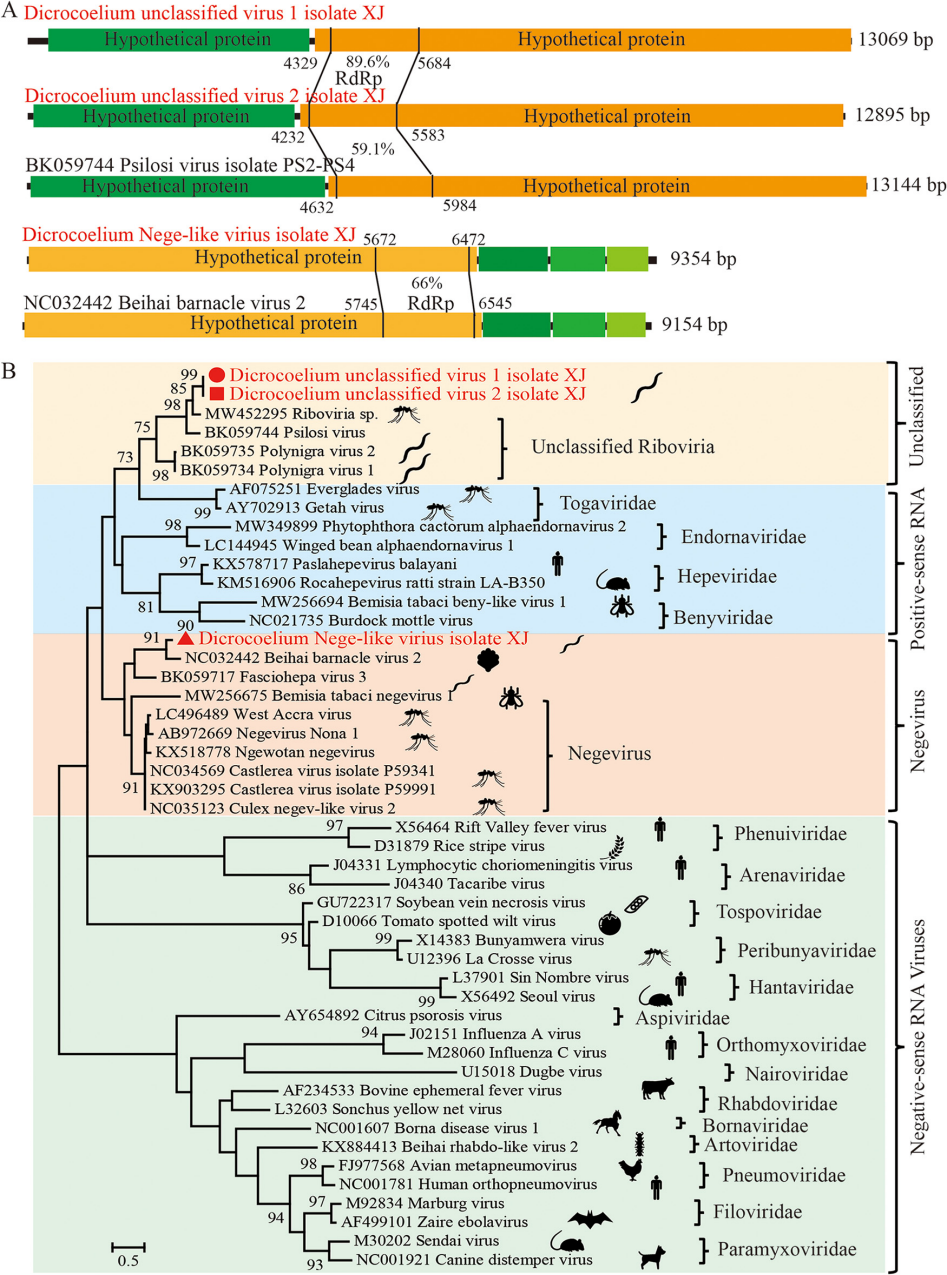
The genomic structures and evolutionary relationships of the newly discovered unclassified RNA viruses with other representative RNA viruses. (A) The genomic structures of unclassified RNA viruses and their closely related homolog. Hypothetical ORFs, the hypothetical protein including the RdRp region, and genetic identities of the RdRp region between unclassified RNA viruses and its homolog are shown. (B) Phylogenetic trees were generated using the full-length coding sequences of the RdRp. MEGA 7 was used for phylogeny inference using the maximum-likelihood (ML) method based on the LG+G model, and the analyses were conducted with 1,000 bootstrap replicates. Representative viruses from different genera of unclassified RNA virus families and the most closely related viruses from the database were included.

In addition to the rhabdo-like virus and endornavirus, we successfully assembled the complete or near-complete genomes of three other RNA viruses. Based on the blastn/r search, their most closely related relatives are all unclassified RNA viruses, indicating three novel RNA viruses that could not be assigned to any current viral families. Next, we selected 2 to 3 representative viruses of all the RNA viral families from the ICTV and constructed a phylogenetic tree using the RdRp regions to investigate the evolutionary relationships of the three novel viruses. The first virus (*Dicrocoelium* Nege-like virus (DiNLV, GenBank no. OP548619)) contains a genome of 9,354 bp and has four main ORFs, similar to its most closely related virus, Beihai barnacle virus 2 (NC032442). These two viruses share approximately 66% identity in the RdRp region and form a close cluster of Negevirus, which belongs to the unclassified ssRNA positive-stranded viruses (Fig. 4A).

The other two RNA viruses have a genome length of 13,069 bp and 12,859 bp. Both viruses (*Dicrocoelium* unclassified RNA virus 1 and 2, GenBank no. OP627659 and OP627660), respectively, encode a 452-amino acid (aa) RdRp protein, and the identity between them is 89.6% (Fig. 4A). Both viruses show high genetic identities to a cluster of viruses recently found in flatworms, with the RdRp identities to these viruses ranging from 43.5% to 64.4% (Fig. 4B). The viruses in this distinct cluster have the same genomic structure, which encodes two large ORFs, and the potential hosts of these viruses are parasitic flatworms, including Psilotrema simillimum and Polycelis nigra (Fig. 4B). Thus, we proposed that this cluster of viruses from different flatworms could be classified as a new family to indicate their evolutionary relationship with others.

### Unclassified CRESS DNA virus.

**FIG 5:**
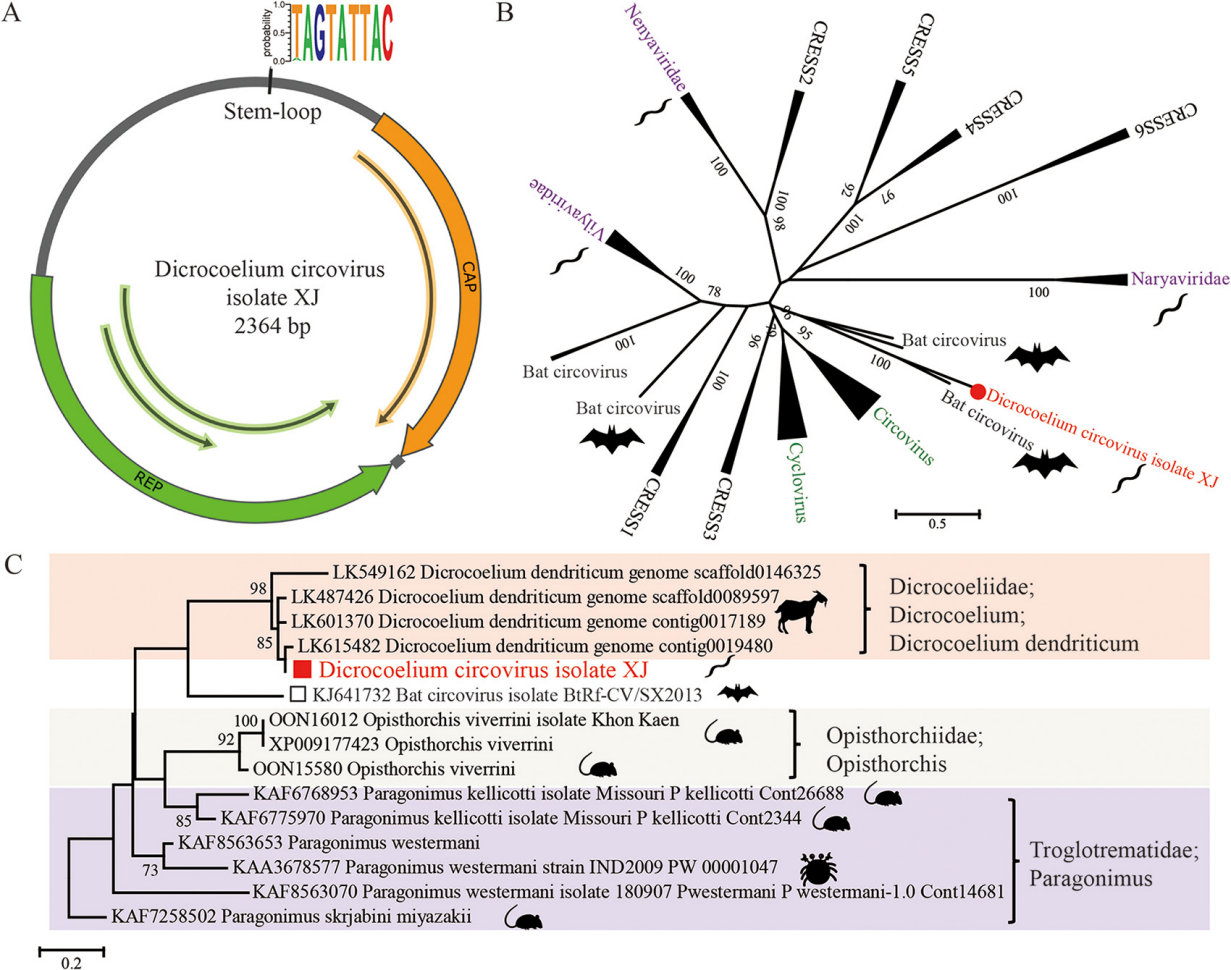
The genomic structure and evolutionary relationships of parasite-associated CRESS DNA virus. (A) The circular genomic structure of DiCV with a length of 2,364 bp; a typical stem-loop (TAGATTAC) is present between Rep and Cap. (B) Evolutionary relationships of DiCV with other representative viruses from CRESS DNA virus. Using the Rep protein, the phylogenetic tree was built using the maximum-likelihood (ML) method under the GTR+G+I model. (C) Evolutionary relationships of the cap protein of DiCV and its most closely related bat circovirus with the genomic sequences of different parasitic flatworms. Phylogenetic analyses were conducted with 1,000 bootstrap replicates.

CRESS DNA viruses are a group of ubiquitous viruses with small circular genomes and a diverse host range (41). In this study, we found a new circular virus from *D. lanceatum*. The genome of this virus is 2,364 bp long and contains the typical Ambisense genome organization and the stem-loop motif TAGTATTAC between the putative capsid (Cap) and replication (Rep) gene (Fig. 5A). The rolling circle replication (RCR) motifs and superfamily 3 (SF3) helicase motifs were identified in the Rep region: RCR motifs I (LVTWNN), II (RHFQC), and III (YCRK); SF3 motifs walker A (FQEESGEL), B (VIDDY), and C (VTSN). This virus is tentatively named *Dicrocoelium circovirus* isolate XJ (DiCV, GenBank no. OQ079367). To further determine its evolutionary relationship with other viruses, we constructed phylogenetic trees using both Rep and Cap sequences from all the representative CRESS DNA viruses. The Rep and Cap genes of this virus are 903 and 675 bp, respectively, and cluster with several bat circoviruses and animal circoviruses. The Rep and Cap sequences show the highest genetic identity of approximately 49.1% and 46.4% to bat circovirus isolate BtRf-CV/SX2013, which was reported in China (KJ641732) (Fig. 5B; see Fig. S1A and B in the supplemental material). Based on a recent study that used a cutoff of 50% Rep protein identity to delimit CRESS virus genera (42), this new virus could be classified into a new CRESS genus.

Interestingly, when the Cap region of DiCV was searched against the NCBI databases (nonredundant nucleotide and protein databases), no virus hit was found. The most closely related sequences were all from whole-genome shotgun contigs of different parasitic flatworms. Then we constructed a phylogenetic tree using the Cap sequence of this virus and genomic sequences of related parasites, and the Cap region was closely clustered with genomic sequences from Dicrocoelium dendriticum, sharing identities ranging from 81.7% to 96.4% (Fig. 5C). These data suggest a novel hypothesis on the origin and evolutionary history of this novel parasite associated circovirus by gaining the Cap region from its host genome or inserting Cap into the host genome.

### Circovirus and cyclovirus.

**FIG 6:**
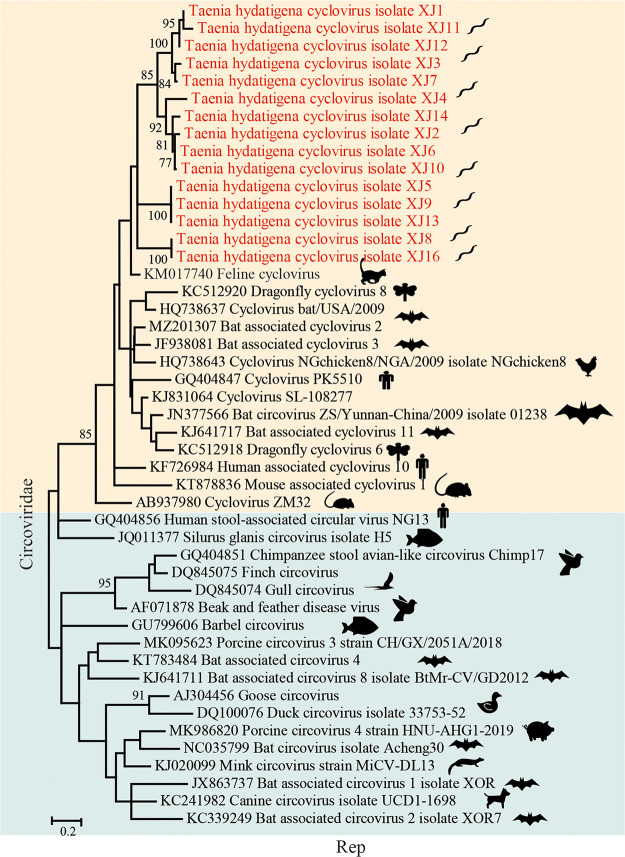
Evolutionary relationships of all cycloviruses and circoviruses found in this study with representative viruses from the *Circoviridae.* Phylogenetic trees were generated using the coding sequences of Rep in the new cyclovirus. MEGA 7 was used for phylogeny inference using the maximum-likelihood (ML) method based on the GTR+G+I model, and the analyses were conducted with 1,000 bootstrap replicates. Representative viruses from different genera of *Circoviridae* and the most closely related viruses from the database were included.

The family *Circoviridae* comprises viruses with circular, covalently closed, single-stranded DNA (ssDNA) genomes, which include the smallest known viral pathogens of animals. Members of the family include circovirus and cyclovirus and infect a wide range of hosts (43, 44). We discovered sequences of a novel cyclovirus in all the *T. hydatigena* samples from 15 dogs, and 15 partial replication-associated protein (Rep) sequences were assembled (GenBank accession no. OP937306 to OP937320). A phylogenetic tree based on Rep sequences shows that all these viral sequences from *T. hydatigena* form an independent cluster (sharing approximately 64.3% to 96.2% identities to each other) and are more closely related (sharing 67.8% to 76.3% identities) to feline cyclovirus (KM017740) (Fig. 6). This viral cluster suggests a new virus species belonging to cyclovirus, which may infect dog-associated *T. hydatigena*.

In addition, a few viral reads related to circovirus were found in one of the *T. hydatigena* pools, and only a 278-bp sequence from the Cap protein was finally assembled (GenBank accession no. OP627662). Phylogenetic analysis based on the partial Cap protein shows that it is highly related (99.6% identity) to canine circovirus (data not shown). Whether the presence of canine circovirus in this parasitic flatworm is due to the ingestion of dog intestinal contents or contamination needs to be further investigated.

### Parvovirus.

**FIG 7:**
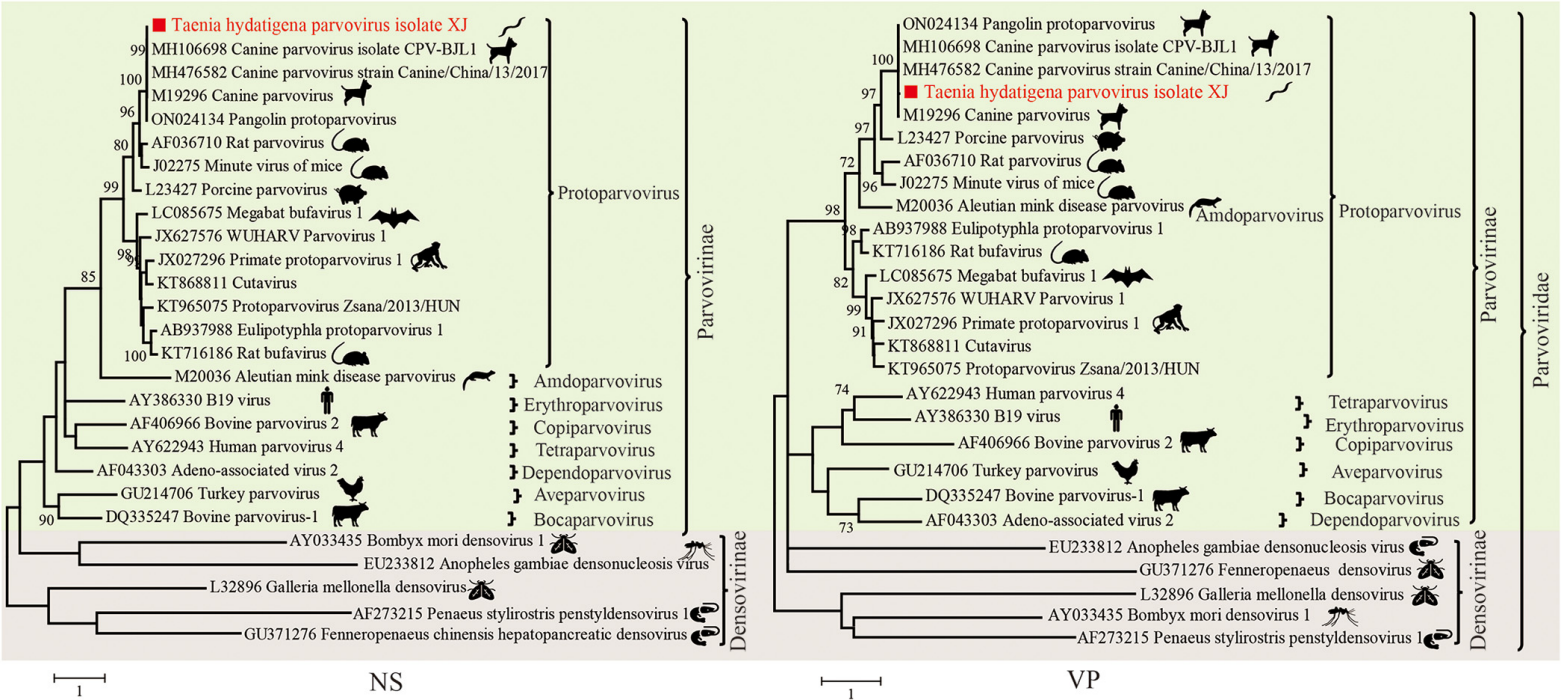
Evolutionary relationships of the parvovirus found in this study with representative viruses from the *Parvoviridae.* Phylogenetic trees were generated based on the full-length coding sequences of the NS and VP regions. MEGA 7 was used for phylogeny inference using the maximum-likelihood (ML) method based on the GTR+G+I model. Analyses were conducted with 1,000 bootstrap replicates. Representative viruses from different genera of *Parvoviridae* and the most closely related viruses from the database were included.

Members of the family *Parvoviridae* are small, nonenveloped viruses with linear, single-stranded DNA genomes of 4 to 6 kb, including *Parvovirinae*, which infects vertebrates, and *Densovirinae*, which infects invertebrates (45). A full-length parvovirus genome of 4,869 bp was assembled from one *T. hydatigena* pool (GenBank accession no. OP627661). Phylogenetic analyses based on both the nonstructural (NS) and viral structural protein (VP) show that the virus clustered with canine parvovirus 2 (CPV2) strains isolated from China (Fig. 7) and displayed high genetic identity (approximately 98.9% at the whole-genome level) to existing CPV2 strains. The presence of a full-length CPV2 genome in the dog-associated *T. hydatigena* could be due to the contamination of dog intestinal contents.

### Verification of the virus using specific PCR assays.

To verify the detection of these diverse viruses using the viral metagenomic method, specific PCR assays were designed based on the assembled viral contigs or genomes. Viral sequences were successfully amplified for all the DNA and RNA viruses (Fig. S2), except for the canine circovirus, which may be due to a low viral load in the sample, as only a few reads were detected. These data confirmed the presence of multiple viruses in these parasitic flatworms.

## DISCUSSION

Parasitic flatworms have long represented a significant economic and health burden due to their high infection rates among humans and domestic animals, including livestock and pets. The Russian doll infection phenomenon in parasitic flatworms has drawn great attention, because parasite-associated microbes could affect the life cycle and fitness of the host parasite and influence the outcome of parasitic infections (46–49). However, the parasite-associated microbiome, including viruses, remains largely unexplored. In this study, we added 10 viral sequences to the database of the parasite-associated viruses, including 8 new viruses, mainly from 2 species of helminths, *T. hydatigena* from dogs and *D. lanceatum* from sheep. The presence of all viruses was confirmed from all parasitic worms using specific PCR assays. These diverse DNA and RNA viruses in the main parasitic flatworms of sheep and dogs highlight the need for large-scale surveillance of the prevalence of the viruses in these animals.

*E. granulosus sensu stricto* and *T. hydatigena* have a similar life cycle between dogs and sheep for harboring adult worms and larval cysts and protoscoleces/cysticercus tenuicollis, respectively. In the same infected sheep, we isolated both cysts/protoscoleces of *E. granulosus sensu stricto* and *D. lanceatum* adult fluke worms. However, viruses were found only in samples from *D. lanceatum*, which indicates a specific parasite rather than a host origin or contamination of these viruses. In addition, no viruses were identified in *E. granulosus sensu stricto* (adult tapeworms) and E. multilocularis (adult tapeworms and protoscoleces) from dogs and mice, and it is possible that the absence of viruses in these tapeworm pools was due to a low prevalence of viruses, and our limited sampling size and/or range may only cover the worms without viruses. A recent study showed that viruses could be vertically transmitted, and they could also be transmitted between different individual worms (23). Thus, future studies are needed to investigate whether the viruses found in *T. hydatigena* and *D. lanceatum* could be detected in their full life cycles and transmitted among different worms as well as the host sheep and dogs.

RNA viruses are ubiquitous and diverse and are likely to exist in all forms of cellular life (2). Our data indicate that similar to other life forms, parasitic flatworms could carry multiple RNA viruses, even though their roles in the host remain unclear. Virus and host codiversification is a common phenomenon, and viruses from closely related hosts are more likely to cluster together in phylogenetic trees (50–53). Several RNA viruses from this study cluster with previously reported parasite-associated viruses. For example, DiRLV1 clusters with several rhabdoviruses found in Clonorchis sinensis and Metorchis orientalis; three unclassified RNA viruses cluster with viruses identified in *P. simillimum* and *Polycelis nigra*. These data indicate that some parasite-associated viruses may have had an independent evolutionary history with their hosts for a long time. In contrast, similar to the previous results, these parasite-associated viruses could also be nested with viruses from other life forms, such as fish and marine arthropods (8), which usually lie in the ancestral positions of phylogenetic trees (54, 55). DiRLV2 is more closely related to the virus found in marine fish; DiRLV1 and an unclassified RNA virus (OP548619) cluster with rhabdovirus and unclassified RNA virus from marine arthropods. One explanation is that the ancient parasites may have already acquired their viruses from marine animal hosts, and after gradual parasite diversification and host switch through different intermediate hosts (50), virus homologs are observed in different current parasitic flatworms and animals, such as sheep (8). Another possibility is that ancient viruses from different animals codiverged into distinct lineages, and a specific parasite may have acquired its virus from its animal host more recently.

The family *Rhabdoviridae* is ecologically diverse, with members infecting plants, animals, including mammals, birds, reptiles, and fish, or invertebrates, including arthropods. Certain viruses from the *Rhabdoviridae*, such as lyssavirus, are deadly to humans and animals. The detection of rhabdovirus in parasitic flatworms raises the question of whether certain parasites could carry or transmit rhabdovirus or other viruses that have potential zoonotic threats and needs further investigation.

Until now, only CRESS DNA viruses have been reported to infect parasitic flatworms, including *Entamoeba* and Giardia parasites (56). The detection of CRESS DNA viruses in *D. lanceatum* further confirms the presence of these small viruses in parasites and broadens their host range. Interestingly, several circular viruses previously detected in bat-associated fecal samples cluster with DiCV, indicating a possible parasite rather than bat origin of these CRESS DNA viruses. Bats are important reservoirs for a diverse range of viruses and other pathogens, including many parasites (57–59); thus, future studies of the bat virome could investigate whether certain viruses are parasite related.

Some viral families, e.g., *Retroviridae*, *Hepadnaviridae*, *Circoviridae*, and *Parvoviridae*, can integrate their viral genomes into host genomes, leaving endogenous viral elements that coevolve with the host (60–62). The high genetic identity and coverage of the DiCV Cap region to the genomic sequences of *Dicrocoelium* suggest the presence of CRESS virus-derived endogenous viral elements in their host genome. This phenomenon was also observed for several other circular viruses that cluster with DiCV, indicating an independent lineage of this virus cluster that coevolves with parasitic flatworms. A possible explanation for the different evolutionary relationships between the Rep and Cap genes could be that the CRESS DNA virus was derived from its host genome, and the host lost its Rep region during the subsequent evolution (63). Another explanation could be that the host gained the virus Cap gene in its genome as a potential defense mechanism (64–66), as the capsid is normally associated with cell entry and host specificity.

Compared with *T. hydatigena*, which was isolated from village stray dogs, no viruses were found in *E. granulosus* and E. multilocularis, which were isolated from laboratory dogs. Stray dogs may have more contact with other animals, especially small mammals, and they consume trash as a food source and contaminated water (67) and may thus have poor health conditions and carry more viruses (68). Even though only partial sequences are available, the presence of the novel cyclovirus in all the individuals of *T. hydatigena* from the intestines of stray dogs supports a potential high host specificity or prevalence of these viruses. The most closely related homolog to this novel parasite-associated virus is a cyclovirus previously reported in cats (69). Several DNA viruses from cats and dogs could be transmitted between them, including parvoviruses (70) and circoviruses (71), which may explain the close relationship between these two circular viruses from different domestic carnivores. We also found the presence of canine circovirus and CPV2 in *T. hydatigena*. These two viruses are associated with many diseases in dogs; however, their origins are unclear, and whether they belong to the intestinal remains during sampling needs to be further clarified.

### Conclusions.

In this study, we characterized the virome of the main parasitic flatworms of major domestic animals and found diverse DNA and RNA viruses from *D. lanceatum* and *T. hydatigena*. Characterizing the parasite virome will greatly expand our understanding of viral diversity, evolution, and ecology. In addition, a viral infection of the parasite can modulate its life cycle and fitness, as well as its pathogenicity, directly or indirectly through interaction with host immunity. The discovery of diverse viruses from important animal parasites highlights the need to investigate the virus-parasite-host interactions, for example, whether viruses can increase the reproduction and transmission of parasitic flatworms or can be responsible for an exacerbated symptom. Animal-derived parasites are a major hazard to animal husbandry; thus, new targeted therapies based on viruses will unravel new strategies to prevent and control parasitic diseases.

## MATERIALS AND METHODS

### Ethics approval.

All the methods and protocols that were used for the infection and isolation of parasites from sheep, dog, and mouse were approved by the Ethics Committee of the First Affiliated Hospital of Xinjiang Medical University (approval no. IACUC-2014021002) and Xinjiang Academy of Animal Sciences (approval no. xjxmkxyAEC20060307).

### Sample processing, library construction, and sequencing.

From May to August of 2021, four species of the main tapeworms in China (*E. granulosus sensu stricto*, E. multilocularis, *T*. *hydatigena*, and *D. lanceatum*) were collected from their corresponding host animals from Urumqi, Xinjiang, China. All samples were stored at −80°C until use. Adult *E. granulosus sensu stricto* and E. multilocularis tapeworms were collected from the intestines of dogs that were artificially infected with protoscoleces of these tapeworms as previously described (72, 73). Briefly, these dogs were orally infected with 1 mL of parasite-precipitated protoscoleces from sheep livers, and the dogs were euthanized by injection of pentobarbital and necropsied 40 days after infection for collecting and counting the adult worms. *E. granulosus sensu stricto* protoscoleces and adult *D. lanceatum* flukeworms were collected from the livers of sheep containing both parasites (confirmed by mitochondrial Cox I and Nad I sequencing; data not shown). E. multilocularis protoscoleces were collected from the liver of intermediate host gerbils (Meriones unguiculatus) that were artificially infected; E. multilocularis protoscoleces were passed by intraperitoneal inoculation of protoscoleces into gerbils every 6 to 10 months in our laboratory. Adult tapeworms of *T. hydatigena* were collected from the intestines of stray dogs in the Midong District of Urumqi, Xinjiang. To avoid potential contamination from the sampling environment, all the worms were first washed three times using sterile phosphate-buffered saline (PBS). Adult worms were first cut into small pieces with scissors, 500 μL PBS was added, and the mixture was homogenized with ceramic beads using a tissue homogenizer (TissueLyser, JINGXIN, China). The homogenates were cleared using centrifugation at 12,000 × *g* for 10 min at 4°C. The resulting supernatant and protoscoleces were extracted using TRIzol reagent (Ambion, USA). RNA was reverse transcribed into cDNA with SuperScript IV reverse transcriptase (Invitrogen, CA, USA) using a random hexamer primer. The second strand of cDNA was generated using the Klenow enzyme (New England Biolabs, MA, USA). Sequencing libraries were constructed using the Nextera XT DNA sample preparation kit (Illumina, CA, USA) and quantified using a Qubit 3.0 instrument (Invitrogen, USA). The quality of each library was checked using Q-sep100 (Bioptic, China) and sequenced on the NovaSeq platform (Illumina, USA) with paired-end (150-bp read length) and dual barcoding for each sample.

### Viral metagenomic analysis.

Virome analysis was performed as previously described (74, 75). In brief, sequencing adaptors and low-quality sequences were removed using Trimmomatic v.0.38 (76). The remaining high-quality reads were *de novo* assembled using Megahit v.1.1.3 (77). Both the singlets and assembled contigs were mapped against a viral nucleic acid database selected from the NCBI nucleotide database (based on annotation taxonomy in the Virus kingdom) using BLASTn (E value, <10^−10^). Reads and contigs with no hits from the previous step were further mapped against a viral protein database with BLASTx (E value, <10^−5^) (DIAMOND v.0.9.24) (78). False positives of the candidate viral hits were removed by mapping all viral hits against the NCBI nucleotide database (ftp://ftp.ncbi.nih.gov/blast/db). The viral reads and contigs were manually checked to exclude potential artifacts.

### Phylogenetic analysis.

To determine the evolutionary relationship of the RNA viruses identified in this study, the RNA-dependent RNA polymerase regions (RdRp) of the six novel viruses and the RdRp sequences from representative RNA viruses (International Committee on Taxonomy of Viruses [ICTV], https://ictv.global/) were extracted and analyzed. The full or partial genomes of each virus were assembled and verified using the Geneious R11 program (79). Viral nucleic acid sequences were first translated into amino acids and then aligned using MEGA7. Alignments were manually checked to avoid mismatches or ambiguous regions. A model test program was used to determine the best substitution model. Maximum-likelihood phylogenetic trees based on nucleotide sequences were generated using the bootstrap method (1,000 times) under the LG+G model or the GTR+G+I model in MEGA 7. Genetic identities between different viruses were calculated based on their nucleotide sequences.

### Detection of newly discovered viruses using specific PCR assays.

Viral nucleic acids were reextracted from all the parasites using the QIAamp virus minikit (Qiagen, Hilden, Germany). Reverse transcription was performed with a random primer with the SuperScript IV reverse transcriptase (Thermo Fisher Scientific). Primers were designed based on assembled viral contigs or genomes, and all the primers used for the amplification of the six new RNA viruses, circular Rep-encoding single-stranded (CRESS) DNA virus, cyclovirus, parvovirus and circovirus are shown in Table S1. The PCR consisted of 15 μL of Thermo Scientific DreamTaq green PCR master mix (2×), 1.5 μL forward/reverse primer in 10 nM, and 1.5 μL of template DNA, and water was added to a final volume of 30 μL. The PCR amplification programs were as follows: denaturation at 95°C for 3 min, 38 cycles at 95°C for 30 s, 55°C for 30 s, and 72°C for 1 min, with a final elongation step at 72°C for 5 min. The PCR results were visualized on 0.8% agarose gel.

### Data availability.

The raw sequencing data were deposited in the Genome Sequence Archive (GSA, https://ngdc.cncb.ac.cn/gsa/) under the BioProject No. PRJNA909433 (BioSample No. SAMN32083958 to SAMN32083977 and SAMN33620605, and SRA No. SRR22558373 to SRR22558392 and SRR23725278). The viral sequences generated in this study can be found at GenBank (accession No. OP548618 to OP548620, OP627658 to OP627662, OP937306 to OP937320, and OQ079367).
