# Supplementary material for: A Diverse Virome Is Identified in Parasitic Flatworms of Domestic Animals in Xinjiang, China
Source: Microbiol Spectr. 2023 Apr 12;11(3):e00702-23. doi: 10.1128/spectrum.00702-23 (PMC10269781; doi:10.1128/spectrum.00702-23)
Supplement: Supplemental file 1 — Supplemental material. Download spectrum.00702-23-s0001.pdf, PDF file, 0.8 MB [file spectrum.00702-23-s0001.pdf]

Table S1. List of the PCR primers that were used for the detection of all the DNA and RNA viruses.

Virus	Target	Designed Primer	Size(bp)
Dicrocoelium circovirus isolate XJ	Cap gene	D-circo-1-F 5’- AACCTCCAATTTCCCTCCGCTA-3’<br>D-circo-1-R 5’- ATTGTCGGGTTGTTCCAGGTCA-3	1673
Dicrocoelium circovirus isolate XJ	Rep gene	D-circo-2-F 5’- GTCTAAACGCTCGCCTCCGAAT-3<br>D-circo-2-R 5’- CGGACGAGTTCCGTAAATGGTG-3	1874
Dicrocoelium unclassified virus 1 isolate XJ	Putative RdRP gene	unclassified virus 1-F 5’- ATGGCAGCGACTGTATTCGA -3’<br>unclassified virus 1-R 5’- CTGCGGCGTCGTAACCTATA -3’	400
Dicrocoelium Rhabdo-like virus 1 isolate XJ	Putative RdRP gene	Rhabdo-like virus 1-F 5’- ACCCCATTTTGGACAGTGACA -3’<br>Rhabdo-like virus 1-R 5’- TCGTGTGCTAATCCCGGATG -3’	400
Dicrocoelium unclassified virus 2 isolate XJ	Putative RdRP gene	unclassified virus 2-F 5’- CGACCGGGATGGCAAGATAT -3’<br>unclassified virus 2-R 5’- TCGACGAACTACGCTTACACG -3’	400
Dicrocoelium Alphaendornavirus isolate XJ	Putative RdRP gene	Alphaendornavirus-F 5’- TTCGTCTGACATGCATTTGGT -3’<br>Alphaendornavirus-R 5’- CGCTCCAATCCCACCAGAG -3’	300
Dicrocoelium Rhabdo-like virus 2 isolate XJ	Putative RdRP gene	Rhabdo-like virus 2-F 5’- ACATCTGGAGCCTCTTTGCA -3’<br>Rhabdo-like virus 2-R 5’- GGTGGGTGCTATTGAGGCTT -3’	400
Dicrocoelium Nege-like virus isolate XJ	Putative RdRP gene	Nege-like virus-F 5’- CAGATATGGCGGGGGTTGTT -3’<br>Nege-like virus-R 5’- TTCTTCTGGAGACGCATCAGT -3’	400
Taenia hydatigena cyclovirus isolate XJ	rep gene	cyclovirus-F 5’- CATAGCACCACCACCGCAA -3’<br>cyclovirus-R 5’- CTCGGGGAGGAAATGGGAATG -3’	350
Taenia hydatigena circovirus isolate XJ	cap gene	circovirus-F 5’- GCCCCATGGTTGACAAGAGG -3’<br>circovirus-R 5’- CCCCCGAATAAACATACGTGACA -3’	260
Taenia hydatigena parvovirus isolate XJ	NS gene	parvovirus-F 5’- AAAGGTGGCGGGCTAATTGT -3’<br>parvovirus-R 5’- GCCAGCCTTGATCTTTTCCC -3’	400

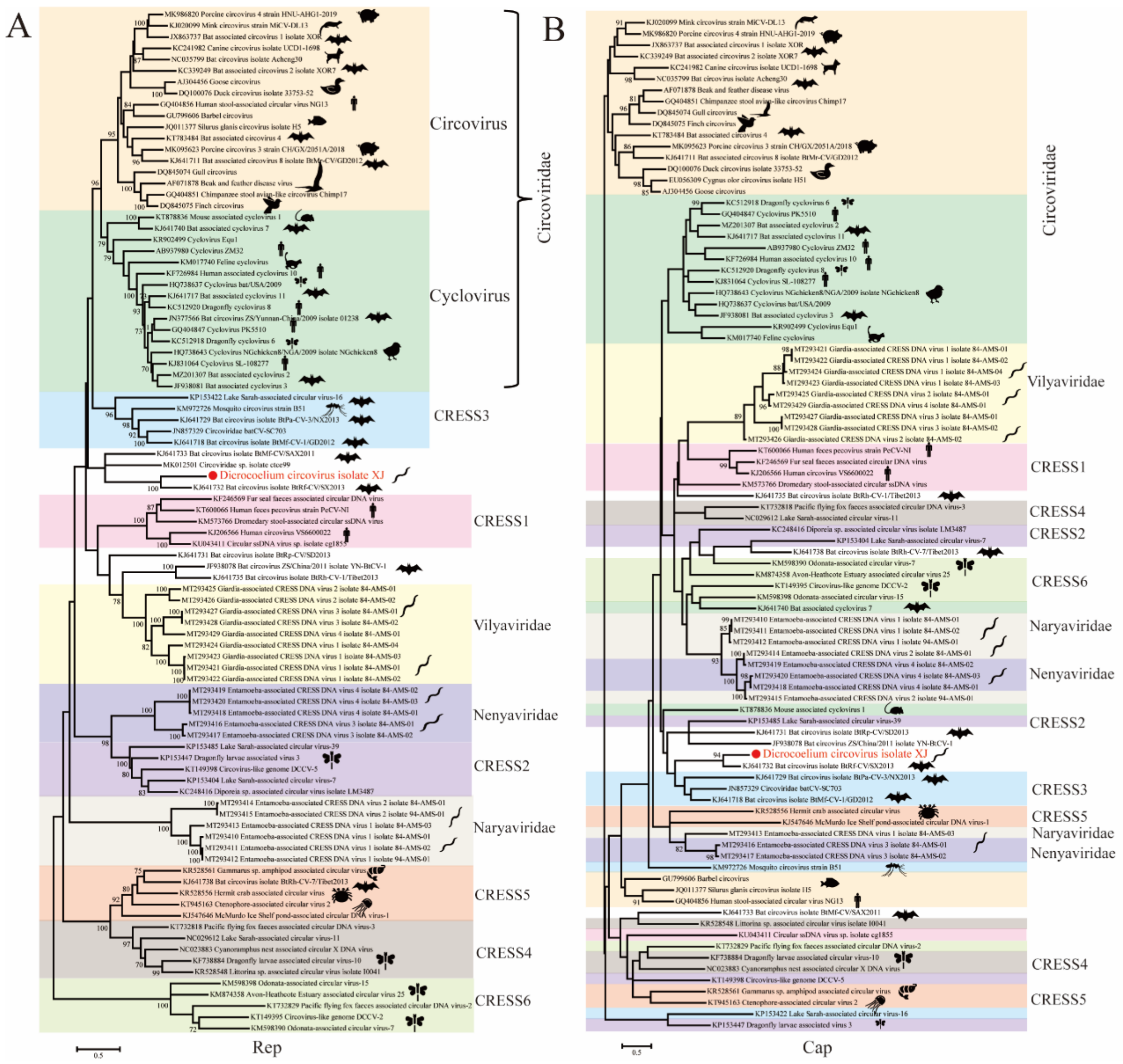

Figure S1 Evolutionary relationships of circovirus found in this study with representative viruses from all CRESS virus.

A: Phylogenetic trees were generated based on full-length coding sequences of rep protein from CRESS virus. B: Phylogenetic trees were generated based on full-length coding sequences of cap protein from CRESS virus. MEGA 7 was applied for phylogeny inference using maximum-likelihood (ML) method based on GTR+G+I model. Analyses were conducted with 1 000 bootstrap replicates. Representative viruses from all CRESS virus and most closely related viruses from the database were included.

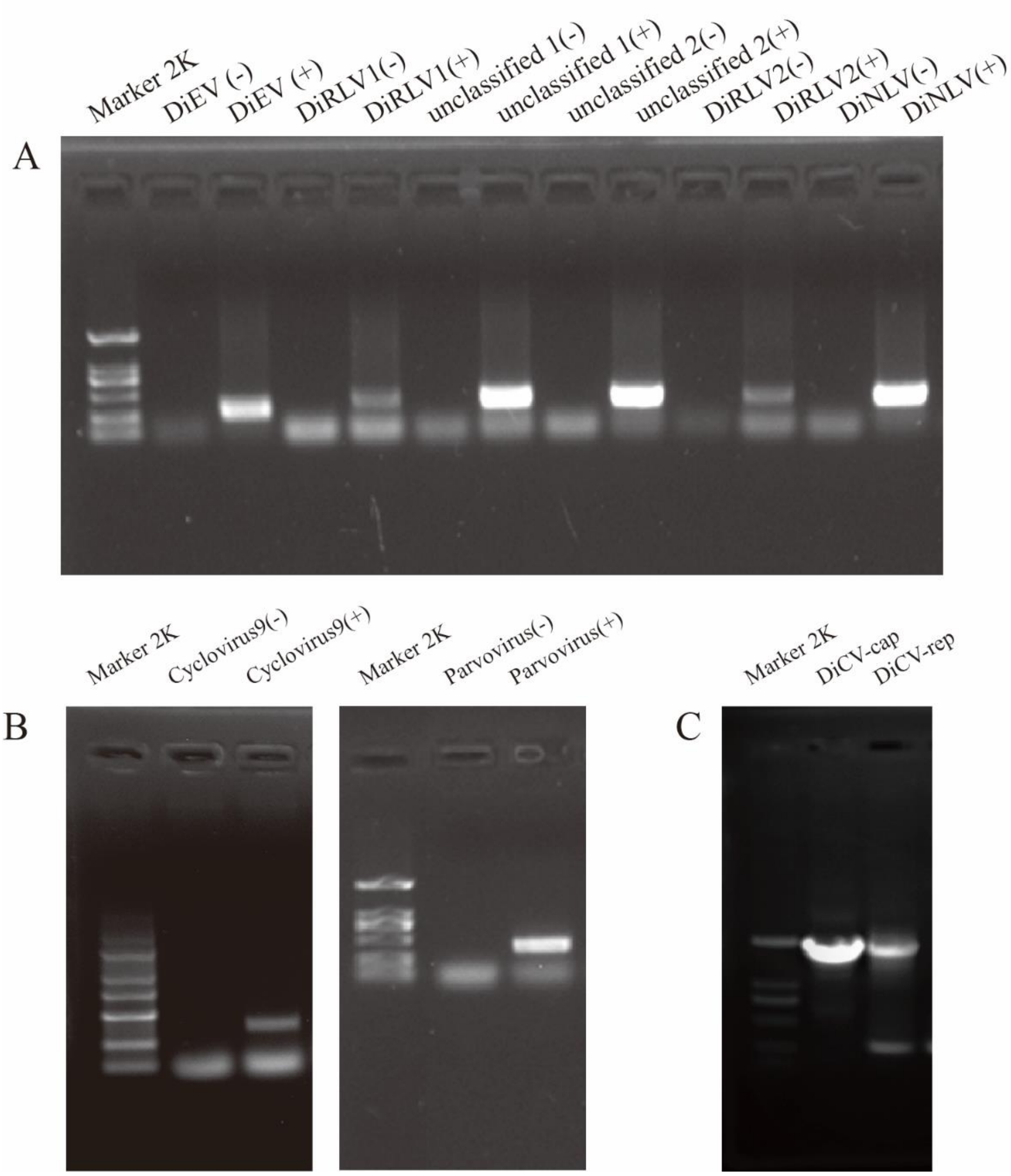

Figure S2 PCR validation results of the new virus in the sample

A: Six RNA virus were amplification in *Dicrocoelium lanceatum* adult tapeworms from sheep liver pool sample, and not in water. The size of product is around 300bp-400bp. B: The *parvovirus* and new *cyclovirus* are amplification in 350bp and 400bp, and not in water. C: The cap and rep for CRESS virus from *Dicrocoelium lanceatum* were amplicated in 1673 bp and 1874bp. The marker of size is 2K.
